# QR code micro-certified gemstones: femtosecond writing and Raman characterization in Diamond, Ruby and Sapphire

**DOI:** 10.1038/s41598-019-45405-7

**Published:** 2019-06-20

**Authors:** Andre Jaques Batista, Pilar Gregory Vianna, Henrique Bucker Ribeiro, Christiano Jose Santiago de Matos, Anderson Stevens Leonidas Gomes

**Affiliations:** 1 Mineral Engineering Department, Universidade Federal de Pernambuco, Recife, Pernambuco Brazil; 2 MackGraphe Graphene and Nanomaterials Research Center, Mackenzie Presbyterian University, 01302-907 São Paulo, Brazil; 3 Physics Department, Universidade Federal de Pernambuco, Recife, 50670-901 Pernambuco Brazil

**Keywords:** Nonlinear optics, Ultrafast lasers, Optical properties of diamond

## Abstract

This paper reports on a micro-certification procedure using femtosecond laser irradiation to microscopically mark a single-crystalline gemological and natural diamond, synthetic ruby and synthetic sapphire, inscribing a QR Code on them. The QR-code was composed of a set of 25 × 25 micropoints, and the irradiation energy was optimized at 1kHz repetition rate. The code was made at a 20 *µ*m relative depth into the gemstone surfaces by controlling the incident laser energy, that was set to 3 μJ for all the samples. Characterization by optical and electron microscopy, as well as micro-Raman hyperspectral imaging showed that the microdots have a diameter of about 14 *µ*m perpendicular to the irradiation direction, being laterally spaced by 14 *µ*m-18 *µ*m applied for each sample. This work corroborates the feasibility of using ultrafast laser inscription technology to fabricate microdots with great quality on gemstone surfaces, which offers a great potential for the jewelry industry to safely micro-encrypt gemological certifications. The compositional and morphological characterization of the modified surface was carried by micro-Raman spectroscopy and scanning electron microscopy.

## Introduction

Diamond, ruby and sapphire are among the most desired and marketable precious gemstones in the world. Due to falsifications (imitations and syntheses) and their great variation in quality and price, certifications are made that try to guarantee their economic, physical and chemical properties. The classic certification is kept on paper and the market needs to re-evaluate the material to see if it corresponds to the one described in the standard. Such work is time consuming and often invasive, requiring the dismantling of the jewelry to remove the stone. Laser written certificates, which have the advantage of being noninvasive, have also been proposed or demonstrated, particularly using bar codes^1–4^.

The technique of femtosecond laser writing (fsLW) in metal, dielectric and semiconductor materials is now a well-established field as recently reviewed in^5,6^, with most of the basic writing mechanisms — which are material dependent — well understood, leading to several scientific and technological applications. Among these materials, the ones studied in this work have already been exploited and diamond is recognized as a photonic platform^7^. In the work of Sotillo^7^, the development of optical techniques to inscribe optical waveguides and other micro-structures in diamond is reviewed. The physical mechanism for fsLW in glasses^5–8^ differs from that in crystals^9,10^, with a local refractive index increase in the former, and an index decrease in the latter.

Quick response codes, more widely known as QR codes, are two-dimensional optically readable bar code systems first employed in the automotive industry in the 1960s, which became worldwide spread with the advent of smartphones^11^. Its applications are countless, from e-commerce to education.

In this work, we report on which is believed to be the first demonstration of gemstone microcertification using femtosecond laser writing to microscopically mark three different gemstones, inscribing a micro QR Code. Natural diamond, synthetic ruby and synthetic sapphire gemstones were used. We anticipate that the best laser parameters for performing the experiment on ruby and sapphire were the same, which was expected as they have the same crystalline structure arising from the corundum mineral^12^.

Further characterization, via micro-Raman spectroscopy, was carried out to verify the composition of the irradiated materials and to better understand the structure of the femtosecond laser-written microdots (see Methods). A scanning electron microscope and a petrographic microscope in reflection and transmitted light mode were used to assist in the analysis of the results.

## Results

**Figure 1:**
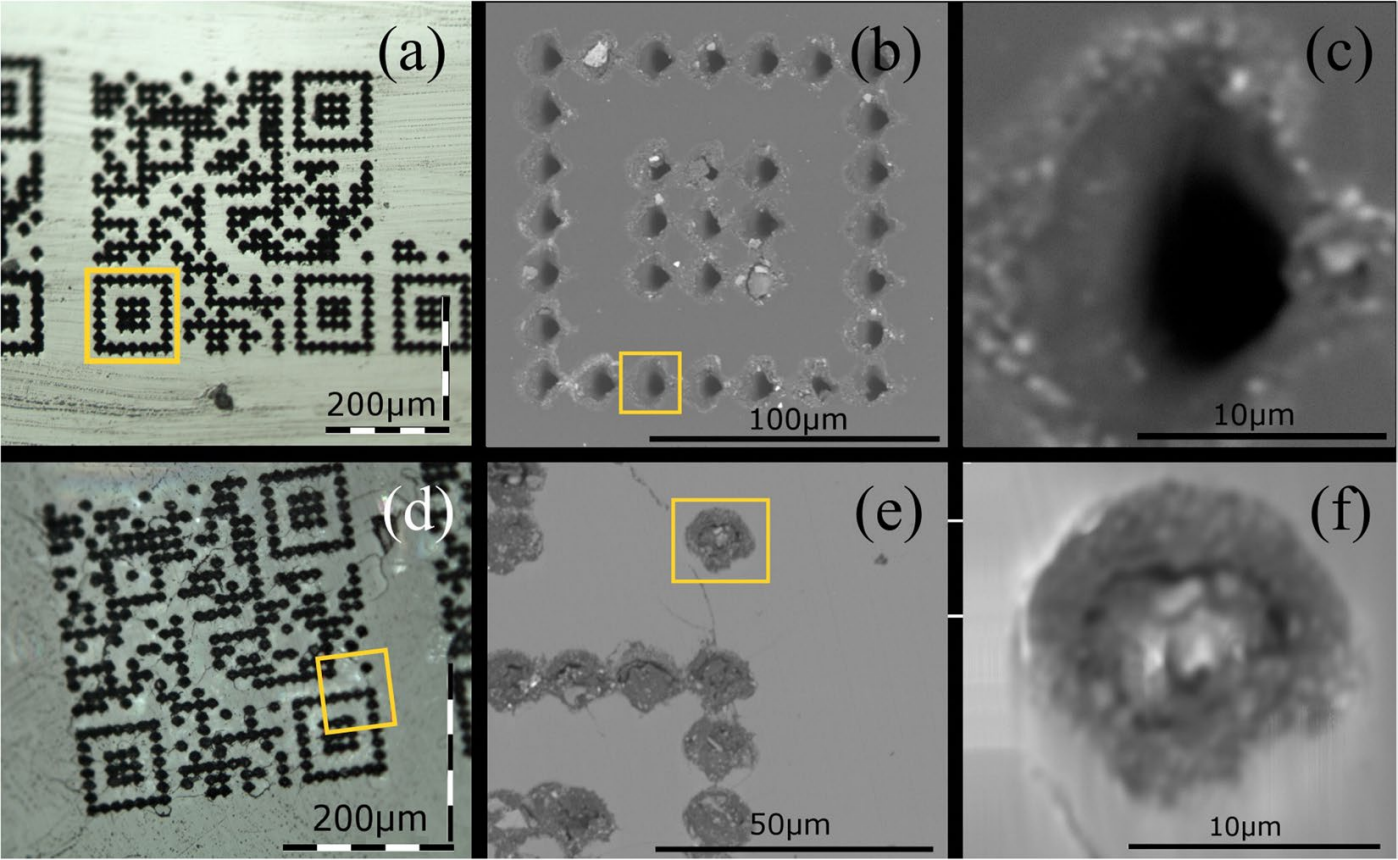
Optical and scanning electron microscopy of the QR codes in diamond (**a**–**c**) and sapphire (**d**–**f**). Yellow squares represent selected area for the SEM analysis shown in the subsequent figure to the right. (**a**) Optical image of the QR code written in diamond obtained with a 100x magnifying set of lenses in the reflection mode. Laser energy was 4 μJ (**b**) SEM image with a magnification of 800x and (**c**) SEM image with a magnification of 7000x. (**d**) Optical image of the QR code written in sapphire obtained in the reflection mode. Laser energy was 3 μJ. (**e**) SEM image with a magnification of 1500x and (**f**) SEM image with a magnification of 8000x.

Figures 1(a,d) show the optical microscope image of the QR-code with 25 × 25 dots, written in diamond and sapphire, forming squares of 432 μm and 336 μm side, respectively. The yellow squares represent selected area for SEM analysis (TM3000 Hitachi tabletop microscope). Figures 1(b,c) show the SEM images of selected regions of the QR code in diamond (yellow square) obtained with magnifications of 800x and 7000x, respectively. Similar data for sapphire are shown in Fig. 1(d–f), with the SEM images for 1500x and 8000x times magnification in Fig. 1(e,f), respectively. Similar results were obtained for ruby (data not shown). For the SEM measurements, low-vacuum operation and charge-up reduction mode were used to avoid the charge accumulation.

**Figure 2:**
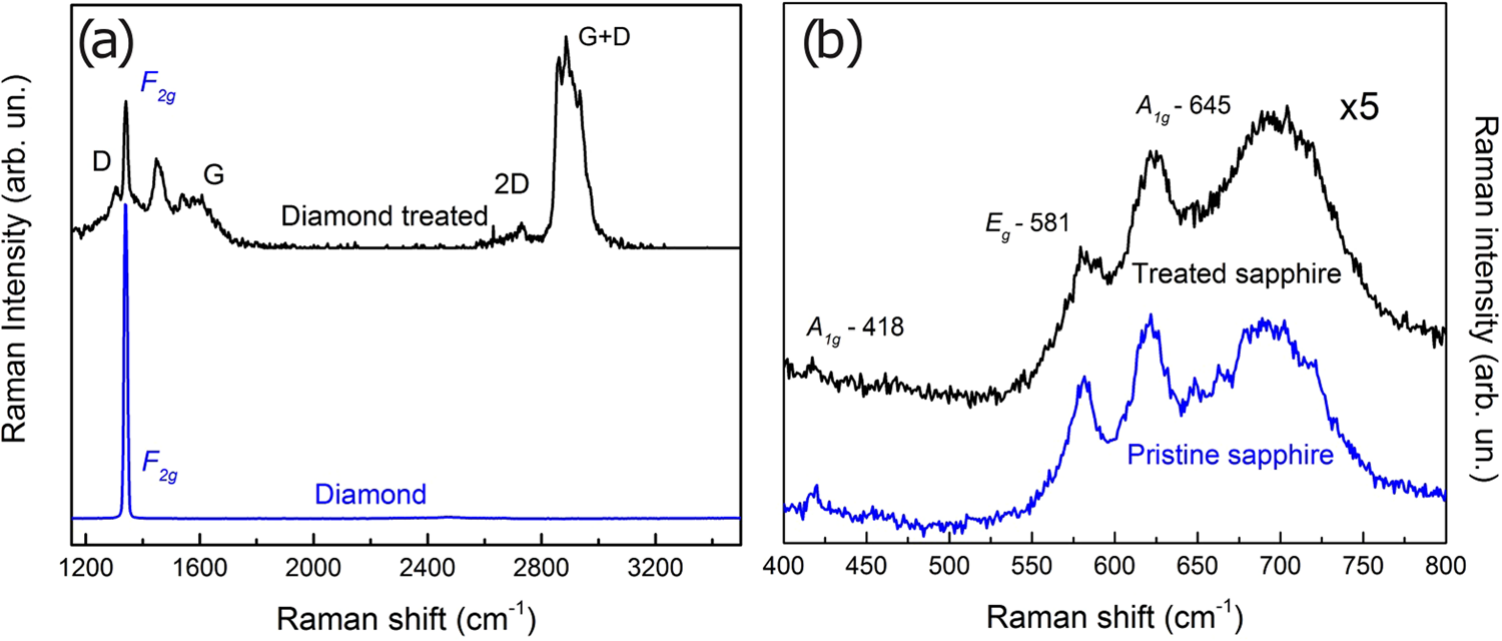
(**a**) Raman spectra of a pristine point on diamond (bottom) compared with a point on the treated part (top). (**b**) Raman spectra of the pristine (bottom) and treated synthetic sapphire (top).

For pristine diamond, the first order Raman spectrum seen in the blue line of Fig. 2(a) shows one characteristic peak centered around 1340 cm^−1^ that can be assigned to the triply degenerated Raman mode F_2g_^13^ related to the presence of sp^3^ bonds in diamond. For sp^2^ carbon structures (e.g., graphite, carbon nanotubes, amorphous carbon) the first order Raman spectrum exhibits a peak at 1560cm^*−*1^, known as the G band, which is attributed to degenerated in-plane transverse and longitudinal optical phonon modes. In addition, defective graphite-like structures exhibit an additional mode centered around 1350cm^*−*1^ known as the D band^14^. These two bands can be seen in the black line spectra shown in Fig. 2(a), corresponding to an irradiated region of the diamond. The appearance of the F_2*g*_ mode together with D and G bands in the black line spectrum suggests the presence of both sp^3^ and sp^2^ bonds in the region where the spectrum was taken and is an evidence of a graphitization that will be discussed later in this work. The graphitization process is also evident in the second order peaks at 2740 cm^*−*1^ (2D peak) and 2940 cm^*−*1^ (D + G peak)^7^.

In the case of sapphire, the spectrum in the untreated area showed three characteristic bands located at 418, 581, 645cm^*−*1^ corresponding to the *α*-Al_2_O-Cr_2_O_3_ phase of corundum, as described by S. P. S. Porto *et al*.^15^ and M. Ashin^16^, shown in the blue line of Fig. 2(b). When the Raman spectrum is measured in a treated point, black line in the figure, the same peaks are observed, but with reduced amplitudes. J. Morikawa *et al*.^17^ makes a correlation of the optical stress with the alteration of the refractive index in sapphire that we can apply to our work, affirming that the parts where a lower Raman intensity peak occurs can indicate (when there is no change of phases nor generation of a new compound) a variation in the refractive index.

**Figure 3:**
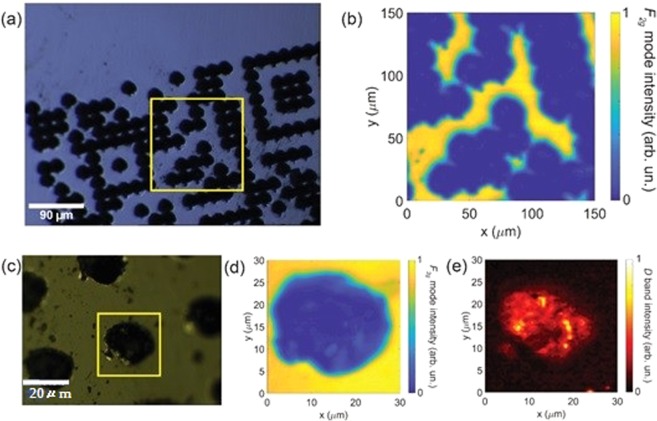
(**a**) Optical image of the femtosecond laser inscribed microdots on the diamond and (**b**) hyperspectral Raman image of the F_2*g*_ mode intensity measured on the area indicated by the yellow square in (**a**). (**c**) Optical image with a higher magnification detailing a single dot. (**d**,**e**) show the hyperspectral Raman images of the F_2*g*_ mode and D band intensities, respectively, measured on the yellow square area indicated in. (**c**) The intensity in all Raman images was normalized by the respective highest value. Color bars indicate the intensity of the modes.

An interesting insight can be obtained by analyzing the hyperspectral Raman images of the pristine and femtosecond laser inscribed microdots in the gemstones. Figure 3(a,c) show the optical images using 10x and 50x objectives respectively, of an inscribed region of the diamond. The hyperspectral Raman image of the F_2*g*_ Raman mode intensity, collected across the yellow squares in Fig. 3(a,c), are shown in Fig. 3(b,d) respectively. Also, Fig. 3(e) shows the hyperspectral Raman image of the D band intensity across the region indicated in Fig. 3(c). The color bar on the right side of each Raman image indicates the corresponding normalized Raman mode intensity. From Fig. 3(b,d) we observe a sharp decrease of the F_2*g*_ Raman mode intensity on the treated region of the crystal. Moreover, from Fig. 3(e) we observe an increase in the D band intensity at the center of the treated region. This is a clear indication of the laser induced graphitization at the center of the mark.

**Figure 4:**
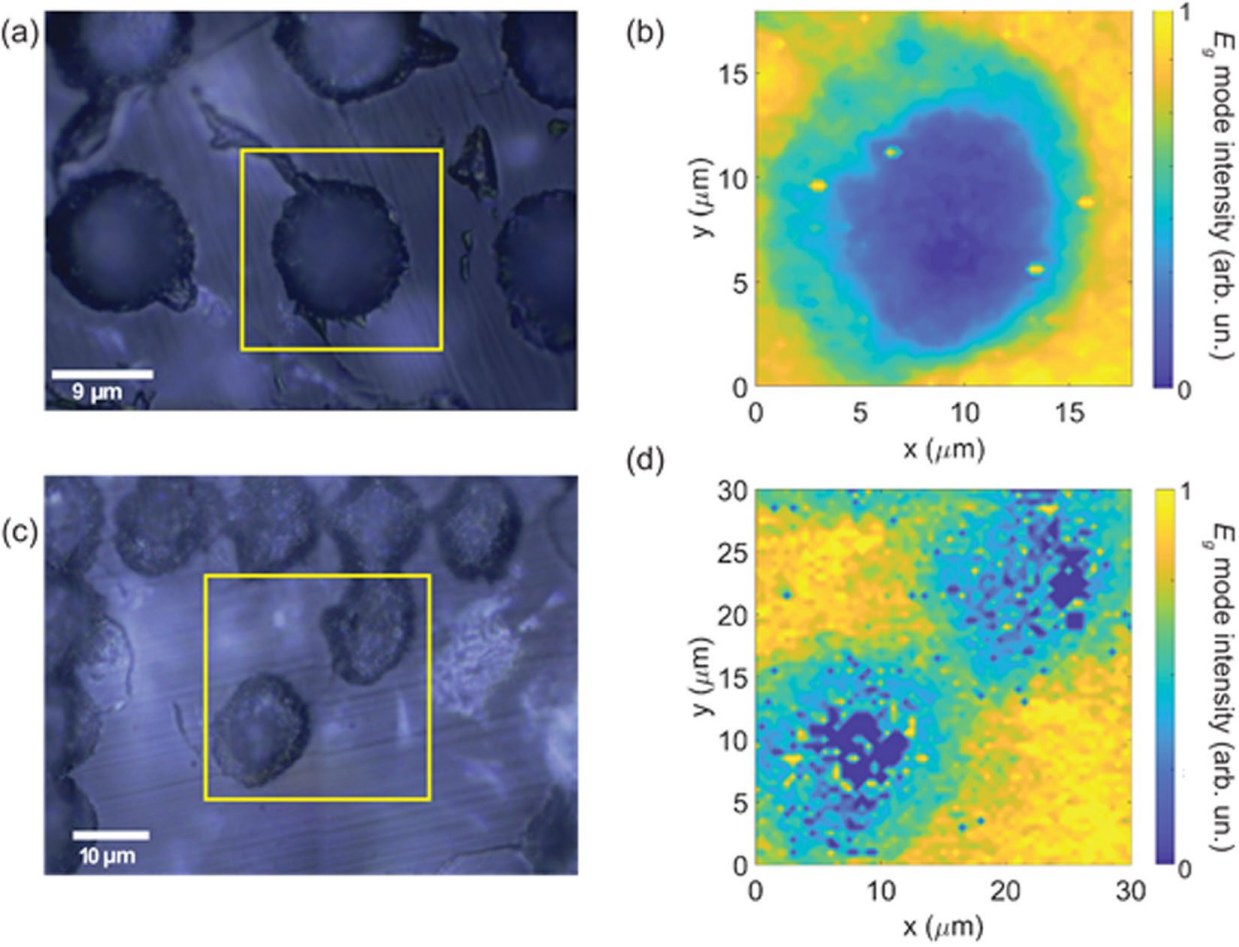
(**a**) Optical image of the femtosecond laser inscribed microdots on the sapphire and (**b**) hyperspectral Raman image of E_*g*_ mode intensity measured on the area indicated by the yellow square in (**a**). (**c**) Optical image of the microdots on the Ruby and (**d**) hyperspectral Raman image of E_*g*_ mode intensity measured in the area indicated by the yellow square in (**c**). The intensity in all Raman images was normalized by the respective highest value. Color bars indicate the intensity of the modes.

Similar measurements were performed in the sapphire and ruby samples and the optical images collected with a 50x objective are shown in Fig. 4(a,c). The hyperspectral Raman image of the E_*g*_ mode intensity, collected across the yellow square of each optical image, is shown in Fig. 4(b,d) for sapphire and ruby, respectively. The normalized intensity of the E_*g*_ mode for each Raman image is indicated by the color bar on the right side of each image. In both cases the hyperspectral Raman images show a decrease in the intensities as one approaches the center of the mark. This effect was discussed by Girolami *et al*.^18^, showing that the higher the laser power, the greater the smoothing of curves in the Raman signal.

## Discussion

It has been recognized that the genesis of femtosecond induced laser modification in glasses, crystals and semiconductors arise from non-linear electron excitation processes occurring due to the ultrahigh intensity of the femtosecond lasers. In dielectrics, multi-photon and tunneling ionization compete to generate free electron plasma. This type of process causes the electrons in the conduction band to absorb the laser radiation very effectively by inverse *bremsstrahlung* and ionize other atoms by collision excitation, causing the plasma formation induced by the intensity dependent Keldysh parameter, as thoroughly reviewed in refs^5,6^. The laser peak power employed in our work was ~30MW, with a 0.17 NA objective, obtained after testing different peak power regimes (for the same microscope objective).

Femtosecond laser inscription and waveguide writing in bulk diamond has been dealt with before^7,19,20^ Sotilo and co-workers^7^ demonstrated that when the repetition rate is reduced from 500kHz to lower values (5 kHz) a greater concentration of nanocrystalline graphite clusters in diamond occurs, corroborating earlier results from the literature^21,22^. Our results for the confocal Raman measurements in Fig. 2(a), as already indicated, further corroborates this explanation. For this reason, a low repetition rate of 1kHz used in the diamond is very appropriate for inscribing QR codes, resulting in a better contrast for the code readout, since the amorphous graphitic carbon shows gray to black appearance, distinguishing well the graphitized points from its unchanged environment. This is, of course, not desirable for light waveguiding, where the loss inherent to the amorphous carbon is detrimental, as already pointed out in^7^. On the other hand, Ionin and co-workers^19^, exploited the so-called supercritical peak power regime, above the self-focusing threshold for diamond, to perform femtosecond laser marking in a spectro-temporal regime for laser writing similar to ours (i.e., fs pulses around 800nm, above the self-focusing threshold). The main difference between the work of ref.^19^ and ours is the repetition rate, which was 10Hz in their case. The physical mechanism follows the same pattern, as well described and reviewed in the literature and referenced here^5–7,19,20^.

As with diamond, femtosecond writing in ruby and sapphire has been demonstrated before^23,24^, but not QR code engraving. The refractive index modification has also been studied before^17^ and our confocal Raman data also corroborate these earlier results. The hyperspectral Raman analysis for all the gemstones studied clearly shows the changes occurring in the irradiated versus nonirradiated areas. The data shows that the crystal modification (graphitization in the case of diamond; Raman intensity reduction in the cases of ruby and sapphire) increases as one approaches the center of the microdots, which is in line with the higher femtosecond laser intensities to which this region was exposed.

For this work, a gemological classification was performed before and after the laser inscription procedure in the materials. Using the current standard of the CIBJO (Confédération International pour la Bijouterie, Joaillerie) and the GIA (Gemological Institute of America), the 4C's are evaluated with a gemological magnifier of 10x magnification and a stoning table^25,26^. It has been determined that a single QR Code inscribed on a gem above 1ct, does not affect any of its "cut" and "clarity" classifications, even with the strict classification for diamonds. Note also that GIA adopts an alphanumeric microinscription in diamonds above 1ct and that can be read with a 10x, or ideally a 20x, magnifying glass. These inscriptions have an average height of 120 μm and a width of 1mm, and are applied to the cut diamond belt. The GIA inscriptions are composed of 3 letters and 10 numbers (e.g, GIA 2116028310). In contrast, our QR code consists of 24 × 24 dots, forming a square with a side length of 432 μm.

In conclusion, the final QR codes inscribed in diamond, sapphire and ruby presented in this work were shown to be functional and are also feasible in other gemstones with some variation of the laser parameters proving the feasibility of using ultrafast laser inscription technology to fabricate microdots with high quality on gemstone surfaces, which offers a great potential for the jewelry industry to microencrypt gemological certifications. QR codes continue to be the subject of investigation against falsifications^27^, and at the same time can be exploited as a noise-free “container” of information^28^. We hope this work opens new ways of mass-producing QR codes by femtosecond inscription.

## Methods

**Figure 5:**
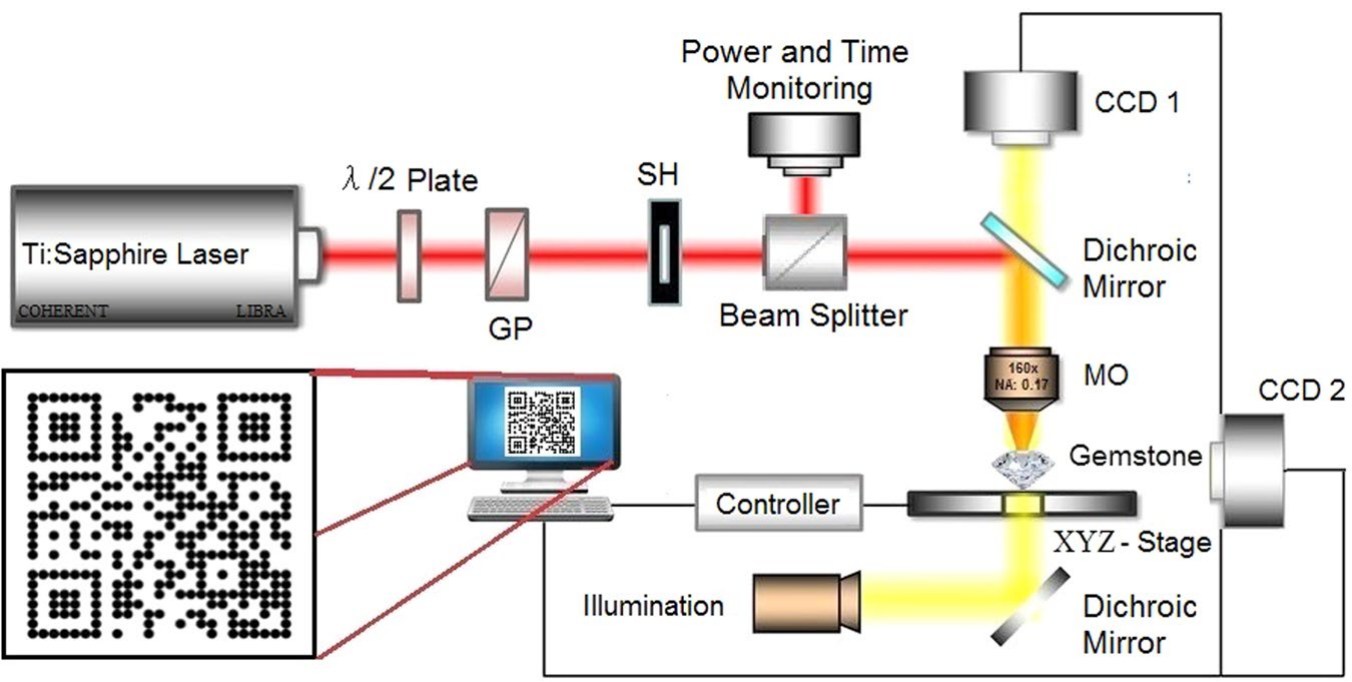
Experimental setup of the femtosecond laser QR Code writing on gemstones and the microscope analyses. Inset (left) shows the custom QR code in dots with redirection to the website www.gemroyalty.com. CCD 01 analyzed the quality of the generated marks. CCD 02 was used to help with the set up of the parameters, such as the relative depth. SH is a shutter. MO is a microscope objetive. GP is a glan prism. BS is a beam splitter.

A standard QR code was produced for use in the tests (inset in Fig. 5), composed of a set of 25 × 25 microdots, which redirects to the website www.gemroyalty.com, made for this project. The QR Code was customized for the dot system and has a mid-level correction rate. The QR code was inscribed in natural diamonds, as well as in synthetic ruby and in synthetic sapphire manufactured by the Verneuil process. All the samples were faceted and polished for jewelry.

Figure 5 shows a diagram of the experimental setup, and the inset shows the designed QR code which is inscribed in the gemstones using the computer controlled XYZ translation stage. The micro QR code was inscribed using a laser system consisting of a Ti-sapphire oscillator followed by a regenerative amplifier (LIBRA Ultrafast Amplifier Laser System, Coherent) delivering 100fs pulses at 800 nm/1 kHz repetition rate, with up to 1mJ energy per pulse, equivalent to an average power of 1W at 1 kHz. The beam was focused into the sample by a 160x microscope objective with numerical aperture of 0.17. The laser intensity was controlled by a half-wave plate followed by a polarizer, and was continuously monitored. The employed energy before the microscope objective was 3 μJ for all the samples, which was the best value achieved after several tests, without damaging the objective. This corresponds to 30 MW laser peak power, which is above the critical peak power for self-focusing (0.4–1 MW), and we are therefore in the so-called supercritical peak power regime, as well discussed in ref.^19^. The inscribed micro dots were characterized by optical microscopy, micro-Raman and scanning electron microscopy. The micro dots were permanently inscribed by using the laser beam focused in a relative depth of 20 μm below the gemstones surface. The beam focus was kept at a fixed position inside the gems, which was translated by a computer controlled 3-axis, 20 nm resolution actuator (Newport Motorized XYZ Translate Stage) in a direction perpendicular to the laser propagation direction after each microdot inscription. The beam was laterally translated by 14 *µ*m for diamond and by 18 *µ*m for sapphire between microdots to inscribe the designed QR code.

Note that, as the depth of focus of the laser beam in the sample may vary depending on the refractive index and the anisotropy of the material to which the laser beam propagates, a relative depth of focus was adopted, where the focus was fixed at the most superficial point of the sample, the beam was blocked and the sample was moved 20 µm towards the lens with the motorized stage, after which the laser beam is released. Therefore, for the same laser parameters and the same material to be processed, reproducibility is guaranteed.

Two CCD cameras monitored the writing process. The CCD 1 camera was part of an optical microscope (Opticam O600P petrographic microscope) operating in reflection and transmission mode with a total magnification between 50X and 1000X, and was used to evaluate, in all stages, the quality of the microdots. Images were collected using an OPT Scientific Cam 16 MP attached to the microscope and two methods of analysis were used. The first method was the analysis of the quality of the point ablation, based on (i) the symmetry and size of the fused, refused and thermal affected areas; (ii) the collateral damage caused by the ejected fused materials; and (iii) the generation of microfractures. The second method was the analysis of the point-to-point ablation quality, that is affected by the interaction between adjacent ablation points. The CCD 2 camera laterally inspected the laser/objective/gem system, and helped in the beam’s initial setup.

A confocal Raman spectrometer (WITec Alpha 300R) using 50x or 10x objectives and 488 nm (2.54 eV) and 532 nm (2.33 eV) laser lines for measurements in diamond, ruby and sapphire was employed. In all cases, the laser power was kept constant at 1mW during measurements. SEM images were made using a scanning electron microscope (SEM; Hitachi tabletop microscope TM3000) with low-vacuum operation and charge-up reduction mode were used to avoid the charge accumulation. Photographs were taken using the Spot FLEX system with Image Capture and Spot software as well as with the SEM.
